# Vaccinia Virus Infection Inhibits Skin Dendritic Cell Migration to the Draining Lymph Node

**DOI:** 10.4049/jimmunol.2000928

**Published:** 2021-01-08

**Authors:** Juliana Bernardi Aggio, Veronika Krmeská, Brian J. Ferguson, Pryscilla Fanini Wowk, Antonio Gigliotti Rothfuchs

**Affiliations:** * Department of Microbiology, Tumor and Cell Biology, Karolinska Institutet, SE-171 77 Stockholm, Sweden;; † Instituto Carlos Chagas, FIOCRUZ, Curitiba PR 81310-020, Brazil; and; ‡ Department of Pathology, University of Cambridge, Cambridge CB2 1QP, United Kingdom

## Abstract

There is a paucity of information on dendritic cell (DC) responses to vaccinia virus (VACV), including the traffic of DCs to the draining lymph node (dLN). In this study, using a mouse model of infection, we studied skin DC migration in response to VACV and compared it with the tuberculosis vaccine *Mycobacterium bovis* bacille Calmette–Guérin (BCG), another live attenuated vaccine administered via the skin. In stark contrast to BCG, skin DCs did not relocate to the dLN in response to VACV. Infection with UV-inactivated VACV or modified VACV Ankara promoted DC movement to the dLN, indicating that interference with skin DC migration requires replication-competent VACV. This suppressive effect of VACV was capable of mitigating responses to a secondary challenge with BCG in the skin, ablating DC migration, reducing BCG transport, and delaying CD4^+^ T cell priming in the dLN. Expression of inflammatory mediators associated with BCG-triggered DC migration were absent from virus-injected skin, suggesting that other pathways invoke DC movement in response to replication-deficient VACV. Despite adamant suppression of DC migration, VACV was still detected early in the dLN and primed Ag-specific CD4^+^ T cells. In summary, VACV blocks skin DC mobilization from the site of infection while retaining the ability to access the dLN to prime CD4^+^ T cells.

## Key Points

- Replication-competent VACV inhibits skin DC migration to draining lymph node.
- The VACV-suppressive effect is a general phenomenon of VACV infection in the skin.
- VACV can access the lymph node in the absence of DC transport and prime CD4^+^ T cells.

## Visual Abstract

**Figure:**
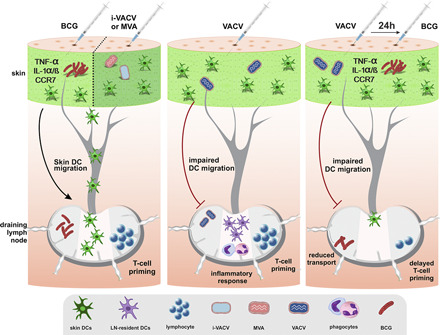

## Introduction

Dendritic cells (DCs) excel in their capacity to capture, transport, and present microbial Ag to prime naive T cells in secondary lymphoid organs (1). The lymph node (LN) is a major site for such Ag presentation, which is often preceded by the relocation of DCs from the site of infection in the periphery to the draining LN (dLN) (2). Despite a large body of data on immunizations with model Ags, DC migration remains incompletely understood during infection with pathogens and live attenuated bacterial or viral vaccines. Using an infection model in mice and a novel assay to track DC migration in vivo, we have previously identified a role for IL-1R signaling in mobilizing skin DCs to the dLN in response to *Mycobacterium bovis* bacille Calmette–Guérin (BCG), the live attenuated tuberculosis vaccine (3). We found that the population of migratory EpCAM^low^ CD11b^high^ skin DCs were important for the transport of BCG from its inoculation site in the skin to the dLN and, in doing so, for priming mycobacteria-specific CD4^+^ T cells in the dLN (3).

Similar to BCG, the smallpox vaccine vaccinia virus (VACV) is a live attenuated microorganism administered via the skin. Despite many studies on the immune response to poxviruses and countless investigations on antiviral T cell priming, there is a knowledge gap on the initial immunological events that unfold in vivo in response to VACV. Because of its large genome and replication cycle features, VACV is readily used as an expression vector and live recombinant vaccine for infectious diseases and cancer (4–7). Because BCG efficacy is suboptimal, there is a standing need to improve tuberculosis vaccination. Recombinant BCG strains as well as novel vaccine candidates are considered or have been developed, some of which are currently undergoing clinical trials. These efforts include attenuated or recombinant VACV vectors and, in fact, modified VACV Ankara (MVA) expressing *Mycobacterium tuberculosis* Ag85A is an example of a clinically-advanced vaccine candidate (8).

Following inoculation of VACV in the skin, infected cells, including DCs and macrophages, can be detected in the dLN within a few hours (9–12). It is not entirely clear if this rapid relocation of virus from skin to the dLN occurs through direct viral access to lymphatic vessels, as also observed after skin infection with Zika virus (12), or if it is supported by other mechanisms. In contrast, other studies indicate that VACV is largely restricted to its inoculation site in the skin, with limited or no relocation of virus to the dLN (13, 14). In this regard, VACV can interfere with fluid transport in lymphatic vessels and, as such, can curb its dissemination (15). In addition to data on viral traffic to the dLN, there is substantive literature on immune evasion and immunosuppression mediated by VACV in vitro and in models of infection (16). Using an established toolset and mouse model for investigating DC responses to mycobacteria we compared local BCG-triggered inflammatory responses in the skin and skin dLN with that of VACV and focused on the ability of VACV to mobilize skin DCs to the dLN. Unlike the early reaction to BCG, we found that VACV actively inhibits skin DC migration to the dLN but retains the ability to enter the dLN in the absence of DC transport and prime CD4^+^ T cells therein.

## Materials and Methods

### Mice

C57BL/6NRj mice were purchased from JANVIER LABS (Le Genest-Saint-Isle, France) and used as wild-type controls. P25 TCR transgenic (Tg) RAG-1^−/−^ mice expressing EGFP (17) were kindly provided by Dr. R. Germain (National Institute of Allergy and Infectious Diseases, National Institutes of Health). Animals were maintained at the Department of Comparative Medicine, Karolinska Institutet. Both male and female mice between 8 and 12 wk old were used. Animals were housed and handled at the Department of Comparative Medicine according to the directives and guidelines of the Swedish Board of Agriculture, the Swedish Animal Protection Agency, and Karolinska Institutet. Experiments were approved by the Stockholm North Animal Ethics Council.

### Mycobacteria

*M. bovis* BCG strain Pasteur 1173P2 was expanded in Middlebrook 7H9 broth supplemented with ADC (BD Biosciences) as previously described (18). Quantification of mycobacterial CFUs for bacterial stocks and determination of bacterial load in LNs was performed by culture on 7H11 agar supplemented with OADC (BD Biosciences).

### Vaccinia virus

VACV Western Reserve (WR) and deletion mutants ΔA49 (19), ΔB13 (20), and ΔB15 (21) (kindly provided by Prof. G. Smith, Cambridge University, Cambridge, U.K.) were expanded on BSC-1 cells. MVA was expanded in BHK-21. Viral stocks were purified by saccharose gradient ultracentrifugation. Quantification of PFUs from WR and focus-forming units (FFUs) from MVA was performed as previously described (22) with MVA stocks quantified in chicken embryo fibroblasts cells, and WR stocks or WR viral load in LNs were quantified on BSC-1. In some experiments, VACV was inactivated with UV radiation (i-VACV) by placing the virus for 2 min in a UV Stratalinker 2400 equipped with 365-nm long-wave UV bulbs (StrataGene). UV inactivation was confirmed by lack of cytopathic effect on BSC-1 cells infected with i-VACV for up to 3 d (data not shown).

Recombinant VACV expressing mycobacterial Ag85B (rVACV-Ag85B) was constructed using the transient dominant selection method (23, 24). Briefly, a cassette containing Ag85B from *M. tuberculosis* (BEI Resources, Manassas, VA) was cloned in a pUC13 plasmid containing the *Escherichia coli* guanylphosphoribosyl transferase (Ecogtp) gene fused in-frame with the EGFP gene under the control of the VACV 7.5k promoter. The correct insertion was confirmed by restriction enzyme digestion and Sanger sequencing. CV-1 cells were infected with WR VACV at a multiplicity of infection of 0.1 PFUs and transfected with the constructed plasmid using TransIT-LT1 in a 2:1 ratio. Progeny virus was harvested after 72 h and used to infect BSC-1 cells in the presence of mycophenolic acid (25 μg/ml), hypoxanthine (15 μg/ml), and xanthine (250 μg/ml). EGFP-positive plaques were selected and purified by three rounds of dilution–infection using BSC-1 cells in the presence of the drugs, as above. Intermediate virus was resolved in BSC-1 cells by three rounds of dilution–infection in the absence of the drugs. The genotype of resolved virus was confirmed to express Ag85B by Sanger sequencing and PCR following proteinase K treatment of infected BSC-1 cells using primers that anneal to the flanking regions of the Ag85B fragment. rVACV-Ag85B was amplified, purified, and quantified in BSC-1, as described above.

### Inoculation of mice

Animals were inoculated in the hind footpad with 30 μl of PBS containing (unless otherwise stated) 1 × 10^6^ CFUs of BCG, 1 × 10^6^ PFUs of VACV, or 1 × 10^6^ FFUs of MVA. i-VACV was used at an amount equivalent to 1 × 10^6^ PFUs before UV-inactivation. Control animals received 30 μl of PBS only. For footpad conditioning experiments, animals were injected in the footpad with PBS, VACV, or i-VACV 24 h before receiving BCG into the same footpad. For studying gene expression in the skin, mice were inoculated in the ear dermis with 5 μl of PBS containing the same concentration of mycobacteria or virus as above. Control animals received 5 μl of PBS.

Assessment of cell migration from the footpad skin to the dLN was done as previously described (3, 25). Briefly, animals previously injected with vaccine or PBS were injected 24 h before sacrifice in the same footpad with 30 μl of 0.5 mM CFSE (Invitrogen). For assessing migration after 24 h, CFSE was injected 2 h after vaccine or PBS inoculation. In control experiments, migration was assessed in response to 1 × 10^5^ PFUs of HSV-1 strain 17 or 100 μg of zymosan (InvivoGen). For studying CD4^+^ Ag-specific T cell responses, 1 × 10^5^ LN cells from naive P25 TCRTg RAG-1^−/−^ EGFP mice were injected i.v. in the tail vein of C57BL/6 recipients in a final volume of 200 μl. Recipients were infected 24 h later in the footpad with 30 μl of BCG or virus. Control animals received PBS. In footpad conditioning experiments, recipients received naive T cells as above and were injected in the footpad 2 h later with PBS, VACV, or i-VACV. BCG was given the next day, and animals were sacrificed 3 and 6 d after BCG, respectively.

### Generation of single-cell suspensions from tissue

Popliteal LNs (pLNs) were aseptically removed, transferred to microcentrifuge tubes containing FACS buffer (5 mM EDTA and 2% FBS in PBS), and gently homogenized using a tissue grinder. The resulting single-cell suspension was counted by trypan blue exclusion. In certain experiments, an aliquot was taken and subjected to CFU or PFU determinations as described above. LN suspensions were otherwise washed in FACS buffer and stained for flow cytometry. Ears were excised, transferred into TRIzol reagent (Sigma-Aldrich), and homogenized in a TissueLyser (QIAGEN) for subsequent RNA extraction, as explained below.

### Flow cytometric staining

Single-cell suspensions from pLN were incubated with various combinations of fluorochrome-conjugated rat anti-mouse mAbs specific for CD4 (L3T4), CD11b (M1/70), CD11c (HL3), MHC-II I-A/I-E (M5/114.15.2), Ly-6G (1A8), Vβ11 (RR3-15) (BD Biosciences), CD326/EpCAM (G8.8), CD103 (2E7) (BioLegend), CD64 (X54-5/7), and CD4 (RM4-5) (eBiosciences) for 45 min at 4°C in FACS buffer containing 0.5 mg/ml anti-mouse CD16/CD23 (2.4G2) (BD Biosciences). Flow cytometry was performed on an LSR II with BD FACSDiva software (BD Biosciences). The acquired data were analyzed on FlowJo software (BD Biosciences).

### Real-time TaqMan PCR

RNA was extracted from ear homogenates and reverse transcribed into cDNA using M-MLV Reverse Transcriptase (Promega). Real-time PCR was performed on an Applied Biosystems PRISM 7500 Sequence Detection System (Applied Biosystems) using commercially available primer pairs and TaqMan probes for TNF-α, IL-1α, IL-1β, CCR7, and GAPDH (Thermo Fisher Scientific). The relative expression of the above factors was determined by the 2^(−△△Ct)^ method, in which samples were normalized to GAPDH and expressed as fold change over uninfected, PBS-injected controls.

### Statistical analyses

The significance of differences in data group means was analyzed by Student *t* test or ANOVA where appropriate, using GraphPad Prism 8 (GraphPad Software) or JMP (SAS Institute), with a cutoff of *p* < 0.05. In some experiments, outliers were excluded from analysis following Grubbs test for outliers (GraphPad).

## Results

### Skin DCs migrate to dLN in response to BCG but not VACV

**FIGURE 1.**
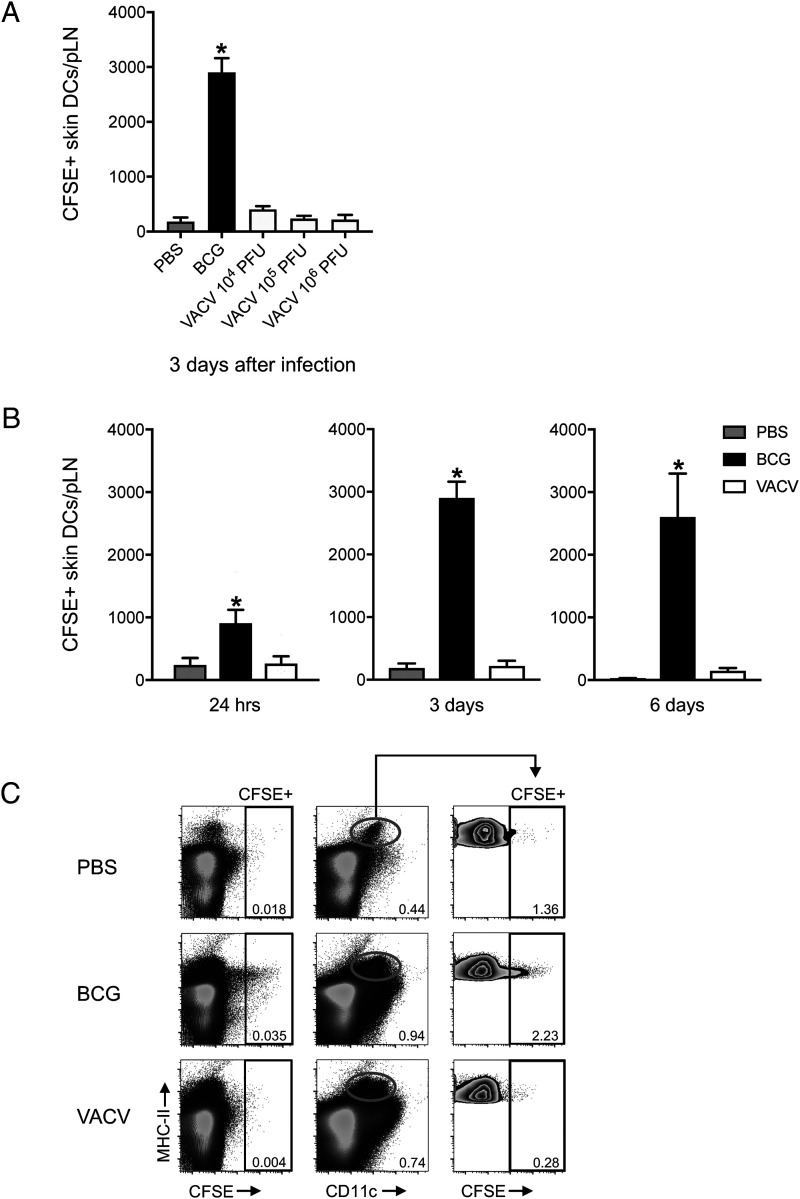
Skin DCs migrate to the dLN in response to BCG but not VACV. C57BL/6 mice were inoculated in the footpad skin with PBS, BCG, or VACV and subjected to the CFSE migration assay (3, 25). Single-cell suspensions were generated from the dLN and pLN and analyzed by flow cytometry. (**A**) Total number of CFSE-labeled skin DCs (MHC-II^high^ CD11c^+/low^) in the pLN 3 d postinfection with 1 × 10^6^ CFUs of BCG or given doses of VACV. (**B**) Total number CFSE-labeled skin DCs in the pLN at the given time points after footpad infection with BCG (1 × 10^6^ CFUs) or VACV (1 × 10^6^ PFUs). (**C**) Concatenated FACS plots showing CFSE-labeled cells from pLN 3 d after PBS, BCG (1 × 10^6^ CFUs), or VACV (1 × 10^6^ PFUs). Representation of CFSE-labeled cells relative to MHC-II (left plots). Skin DCs were gated (center plots), and CFSE-labeled cells are shown (right plots). Four to five animals per time point and group were used in each experiment. One of two independent experiments is shown. Bars indicate SEM. The asterisk (*) denotes statistical difference between PBS and BCG.

To investigate DC migration in response to VACV, we inoculated the virus in the footpad skin of C57BL/6 wild-type mice and used a CFSE fluorochrome-based migration assay to track the movement of skin DCs to the dLN and pLN (3, 25). We have used this setup in the past to study early responses to BCG, another live attenuated vaccine given via the skin and so included BCG in this study as a comparison with VACV. In line with our previous results (3), BCG footpad infection triggered migration of skin DCs to the dLN. However, in stark contrast to BCG, skin DCs did not relocate to the dLN in response to VACV (Fig. 1). The lack of DC movement in response to VACV was independent of viral inoculation dose (Fig. 1A) and the time point at which DC migration was investigated (Fig. 1B). Interestingly, the absence of CFSE labeling in skin DCs in the dLN of VACV-infected mice was not concurrent with CFSE labeling in other MHC class II (MHC-II)^+^ cells or even in MHC-II–negative populations, suggesting a generalized absence of cells moving from skin to the dLN in response to the virus (Fig. 1C).

**FIGURE 2.**
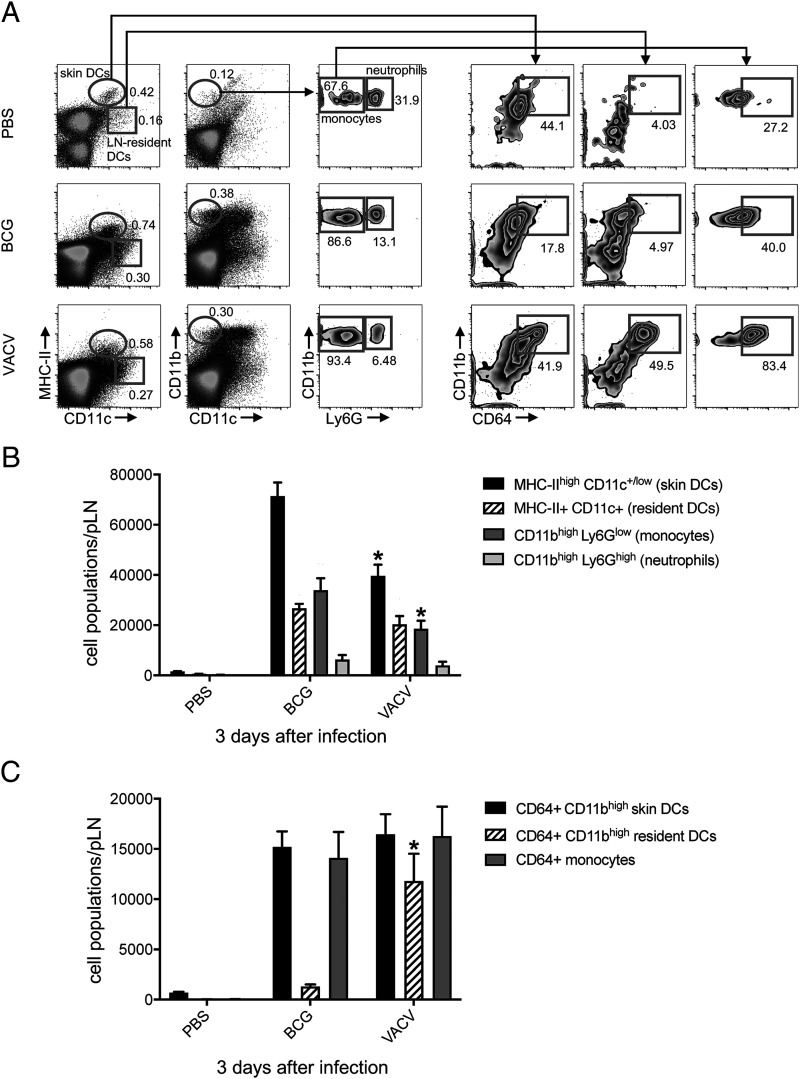
Expansion of phagocyte populations in the dLN after BCG and VACV infection in the skin. C57BL/6 mice were inoculated in the footpad skin with PBS, BCG (1 × 10^6^ CFUs), or VACV (1 × 10^6^ PFUs). Three days later, single-cell suspensions were obtained from the pLN and analyzed by flow cytometry. (**A**) Representative FACS plots showing frequency of different phagocyte populations in each group. (**B** and **C**) Graph showing total numbers for populations in (A). Five animals per group were used in each experiment. Bars indicate SEM. The asterisk (*) denotes statistical significance between BCG and VACV groups (B and C). BCG and VACV populations in (B) and (C) are statistically significant from PBS controls.

Although migratory skin DCs did not readily relocate to the dLN in response to VACV, the infection did provoke a robust inflammatory response in the dLN, expanding several phagocyte populations (Fig. 2A, 2B). The lack of skin DC migration recorded in our CFSE assay was in line with a marked decrease in the overall number of skin DCs (MHC-II^high^ CD11c^+/low^ cells) found in VACV-infected LN, suggesting a major negative impact of virus infection on these cells (Fig. 2B). We also observed high surface expression of CD64 on monocytes and CD11b^high^ LN-resident DCs in the VACV-infected group (Fig. 2A), in which CD11b^high^ LN-resident DCs expressing CD64 were clearly expanded compared with BCG (Fig. 2A, 2C).

### VACV actively inhibits skin DC migration to dLN

**FIGURE 3.**
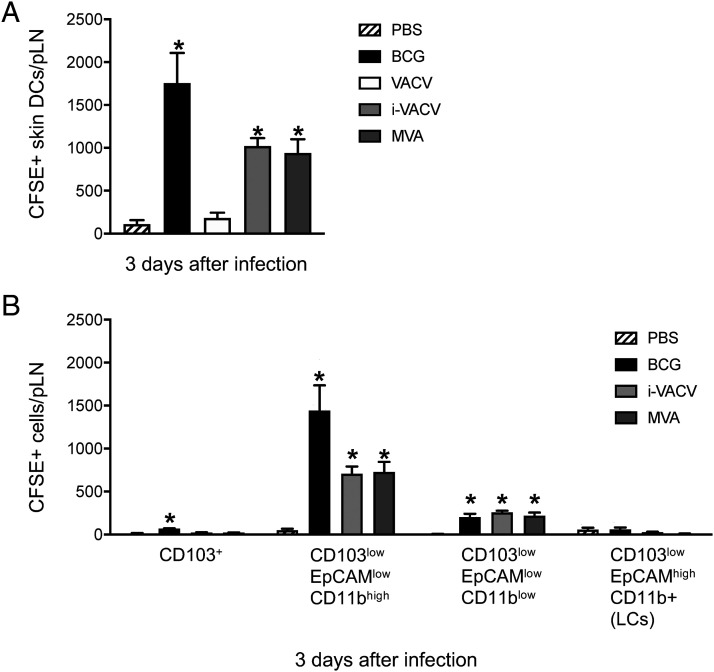
Skin DCs migrate to dLN in response to i-VACV and MVA. C57BL/6 mice were inoculated in the footpad skin with PBS, BCG (1 × 10^6^ CFUs), VACV (1 × 10^6^ PFUs), i-VACV (equivalent to 1× 10^6^ PFUs before inactivation), or MVA (1 × 10^6^ FFUs) and subjected to CFSE migration assay as in Fig. 1. dLN and pLNs were analyzed by flow cytometry 3 d postinfection. (**A**) Total number of CFSE-labeled skin DCs is shown. (**B**) CFSE expression within defined subsets of skin DCs based on previously published gating strategy (3, 25) are shown. Four to five animals per group were used in each experiment. One of two independent experiments is shown. Bars indicate SEM. The asterisk (*) denotes statistical significance between PBS- and vaccine-injected groups. LCs, Langerhans cells.

Because many of the known immunomodulatory molecules produced by VACV require viral replication, we investigated whether the absence of DC migration in response to VACV was coupled to productive infection. We thus exposed VACV to UV cross-linking at levels sufficient to ablate viral replication but without abolishing viral entry into cells (26). Interestingly, inoculation with i-VACV in the footpad promoted skin DC mobilization to the dLN (Fig. 3A). Similarly, skin infection with MVA, a highly attenuated VACV lacking numerous immunomodulators that infects but fails to assemble new virions in mammalian cells (27), also triggered DC migration to the dLN (Fig. 3A). These results indicate that replication-competent VACV actively blocks skin DC migration. Similar to BCG (3, 28), EpCAM^low^ CD11b^high^ DCs were the main DC subpopulation migrating in response to i-VACV and MVA (Fig. 3B).

**FIGURE 4.**
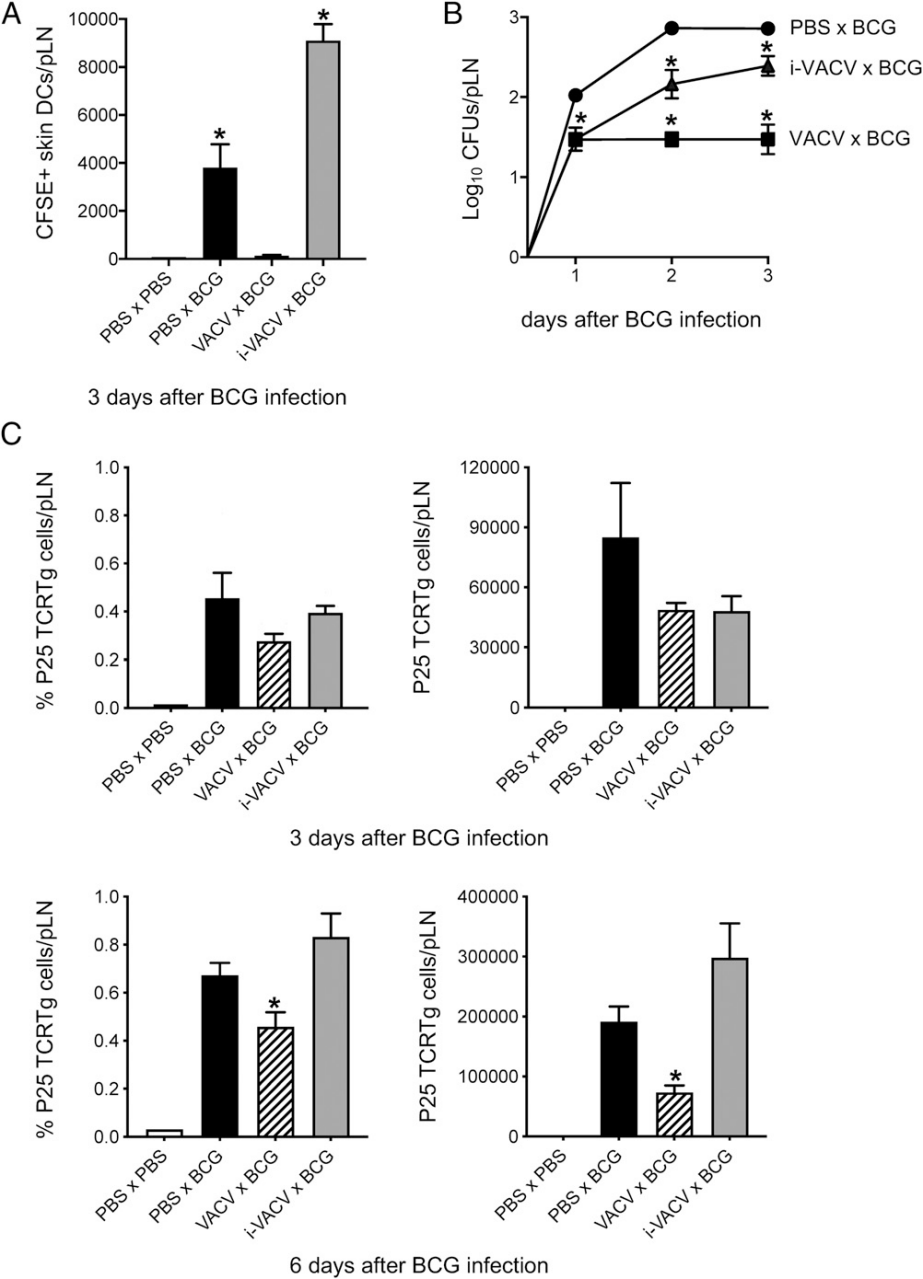
Conditioning the BCG injection site in the skin with VACV mutes DC responses to BCG. C57BL/6 mice were inoculated in the footpad skin with PBS, VACV (1 × 10^6^ PFUs), or i-VACV (corresponding to a dose of 1 × 10^6^ PFUs before inactivation). Twenty-four hours later, the same footpads were inoculated with BCG (1 × 10^6^ CFUs), and the CFSE-based migration assay was performed. (**A**) Total number of CFSE-labeled skin DCs in the pLN 3 d after BCG. (**B**) Recovery of BCG CFUs from the pLN after conditioning with VACV. Before giving BCG, footpads were inoculated 24 h earlier with PBS (PBS × BCG), VACV (VACV × BCG) or i-VACV (i-VACV × BCG). (**C**) Frequency and total number of P25 TCRTg cells in the pLN 3 and 6 d after BCG. Naive P25 TCRTg cells were given i.v. to C57BL/6 recipients, which were then conditioned as in (B). Three and 6 d after BCG, pLNs were processed, and P25 TCRTg cells (EGFP^+^ Vβ11^+^ CD4^+^) were analyzed by flow cytometry. Five animals per group were used in each experiment. One of two independent experiments is shown. Bars indicate SEM. The asterisk (*) denotes statistical significance between PBS × PBS controls and vaccine-injected groups (A); between PBS × BCG and i-VACV × BCG or VACV × BCG groups (B); between PBS × BCG and VACV × BCG groups (C). Expansion of P25 TCRTg cells is statistically significant in conditioned groups relative to PBS × PBS controls (C).

Consistent with a potent suppressive effect on skin DC migration, conditioning the footpad with VACV prior to injecting BCG in the same footpad completely blocked skin DC migration to BCG (Fig. 4A). As expected, the absence of skin DCs entering the dLN was associated with a massive reduction in BCG detected in the dLN (Fig. 4B). DC migration was not impaired when conditioning was done with i-VACV (Fig. 4A). Rather, the number of moving DCs was increased in the i-VACV–conditioned group (Fig. 4A), although BCG entry itself was slightly lower in this group compared with controls conditioned with PBS instead of virus (Fig. 4B). In line with muted DC responses and BCG transport to the dLN, VACV conditioning also delayed the priming of mycobacterial Ag85B-specific P25 TCRTg cells in the dLN (Fig. 4C). Overall, these experiments suggest a carry-over of the DC migration-dampening properties of VACV to a secondary challenge with BCG.

### Enhanced mRNA expression of inflammatory mediators associated with BCG-triggered DC migration is absent from the skin of VACV-infected mice

**FIGURE 5.**
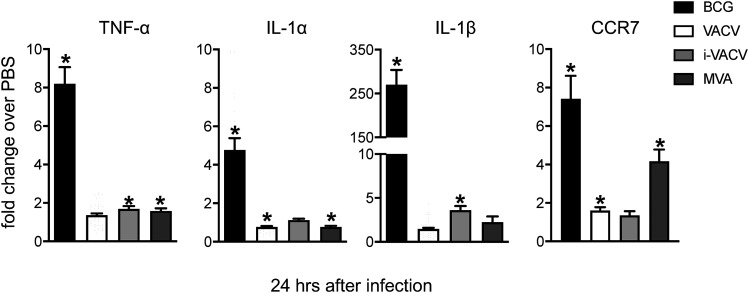
Enhanced mRNA expression of proinflammatory mediators associated with BCG-triggered DC migration are absent from virus-infected skin. C57BL/6 mice were inoculated intradermally in the ear with PBS, BCG (1 × 10^6^ CFUs), VACV (1 × 10^6^ PFUs), i-VACV (equivalent to 1 × 10^6^ PFUs before inactivation), or MVA (1 × 10^6^ FFUs). Ears were removed 24 h postinfection and subjected to RNA extraction and cDNA synthesis. The mRNA accumulation of TNF-α, IL-1α, IL-1β, and CCR7 relative to GAPDH was determined by real-time TaqMan PCR, and the fold change of infected animals over PBS controls was calculated. Data pooled from three independent experiments (including 15–38 samples per group) are shown. Bars indicate the SEM. The asterisk (*) denotes statistical significance between PBS- and vaccine-injected groups.

Next, we compared local changes at the site of infection following inoculation with either vaccine. Transcription of the proinflammatory cytokines TNF-α, IL-1α, and IL-1β was clearly detected in the skin 24 h after BCG infection (Fig. 5), corroborating our previous data on a role for IL-1R signaling in regulating DC migration to BCG (3). On the contrary, enhanced expression of TNF-α and IL-1 was absent in response to VACV (Fig. 5). Interestingly, the same was also observed in response to i-VACV and MVA (Fig. 5). Furthermore, expression of the LN-homing chemokine receptor CCR7 was high in the skin postinfection with BCG and MVA but not VACV or i-VACV (Fig. 5), suggesting differences between i-VACV and MVA, although both trigger skin DC migration.

### VACV is detected early in dLN postinfection in the skin and primes Ag-specific CD4^+^ T cells

**FIGURE 6.**
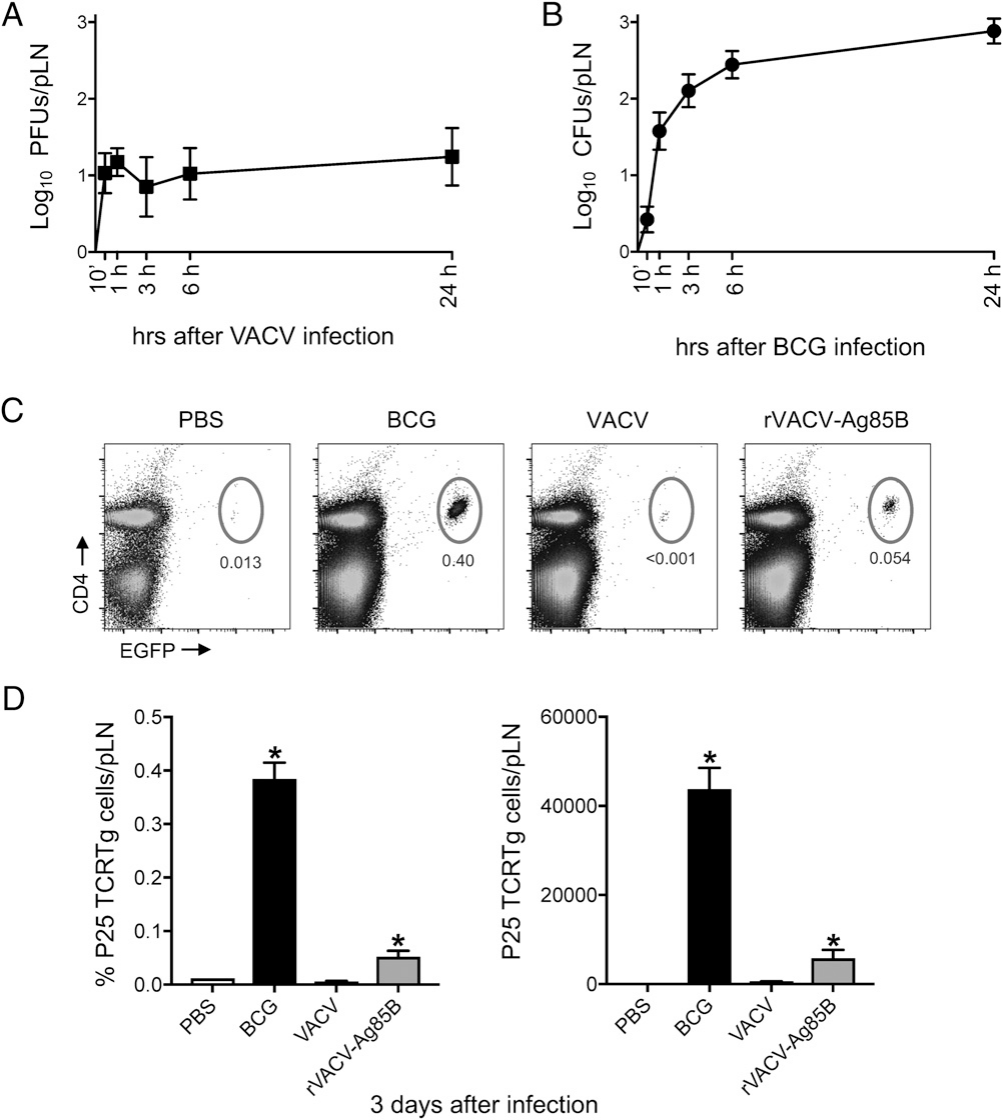
VACV is detected early in dLN postinfection in the skin and leads to priming of CD4^+^ T cells. (**A** and **B**) C57BL/6 mice were inoculated in the footpad skin with VACV (1 × 10^6^ PFUs) or BCG (1 × 10^6^ CFUs). Viral (A) and mycobacterial (B) loads in pLN were determined at different time points postinfection. (**C** and **D**) Frequency of P25 TCRTg cells in pLN after footpad infection with BCG or rVACV-Ag85B. Naive P25 TCRTg cells were given i.v. to C57BL/6 recipients, which were infected 24 h later in the footpad skin with PBS, BCG (1 × 10^6^ CFUs), VACV (1 × 10^6^ PFUs), or rVACV-Ag85B (1 × 10^6^ PFUs). Three days postinfection, pLNs were processed, and P25 TCRTg cells (EGFP^+^ Vβ11^+^ CD4^+^) were analyzed by flow cytometry. Five animals per time point and group were used in each experiment. One of two independent experiments is shown. Bars indicate SEM. The asterisk (*) denotes statistical significance between PBS- and vaccine-injected cohorts.

Although DC migration was blocked in response to VACV, the virus was detected in the dLN as early as 10 min postinfection in the footpad skin, and levels remained steady over time (Fig. 6A). The kinetics of this response was different and notably faster than the entry of BCG into the dLN (Fig. 6B), which is reliant on DC transport (3). To compare priming of CD4^+^ T cells to VACV and BCG using the same tool, we engineered rVACV-Ag85B, and used P25 TCRTg cells to gauge T cell priming in the dLN after footpad infection. BCG and rVACV-Ag85B but not VACV expanded P25 TCRTg cells in the dLN (Fig. 6C, 6D). This reveals that the amount of VACV that relocates to the dLN in the absence of DC transport is clearly sufficient to expand Ag-specific CD4^+^ T cells in the dLN.

## Discussion

VACV infects a variety of cell types in the skin, including keratinocytes and epidermal and dermal DCs (29). The virus is intriguing, given that it carries a diverse immunosuppressive arsenal but remains highly immunogenic. Concurrent with this complexity, the outcome of DC–VACV interactions remains incompletely understood. The fate of the virus and its transport to the dLN for Ag presentation are matters of interest, given that attenuated or recombinant VACV is often considered as a vaccine vector, oncolytic agent, and tool for Ag delivery. We report that VACV profoundly inhibits the ability of skin DCs to mobilize to the dLN. This inhibition is dependent on viral replication and capable of dampening DC migration to a subsequent challenge with BCG. VACV can nevertheless relocate to the dLN in the absence of DC mobilization and prime CD4^+^ T cells. Our study supports recent observations that LN conduits transport VACV to the dLN for T cell priming (12). We also add to a large body of data on the immunosuppressive properties of VACV (16) and extend these to include virus-mediated inhibition of skin DC migration.

VACV can infect DCs but undergoes abortive replication (30–33). That said, VACV infection is known to have many negative effects on DC function. For instance, the virus can inhibit expression of DC costimulatory molecules and cytokines (30, 32, 33). Splenic DCs isolated from VACV-infected mice express less MHC-II and have lower Ag presentation capacity (34). We find lower expression of MHC-II on migratory skin DCs from the dLN of both VACV- and BCG-infected mice (Supplemental Fig. 1A). VACV and MVA can induce apoptosis in DCs (34). Although we did not investigate virus-induced DC death in our studies, the frequency of migratory skin DCs in the dLN of VACV-infected mice was similar to that of PBS-injected controls (Fig. 2A, Supplemental Fig. 1B). This speaks against massive DC death in the skin, which would lower the availability of migratory DCs in the skin and consequently the frequency of these DCs in the dLN. Results from our migration assay point instead to an impediment of skin DC traffic to the dLN during VACV infection.

Both VACV and MVA have previously been shown to inhibit migration of human monocyte-derived DCs toward CCL19 by interfering with CCR7 signaling rather than surface expression of the receptor (35). Although we did not formally investigate this in our model, inhibition of CCR7 signaling by VACV could be a possible explanation for impaired DC migration. Indeed, we observed muted influx of skin DCs and BCG into the dLN when the injection site in the footpad skin was preconditioned with VACV. Thus, the suppressive effect of VACV on DC migration was robust enough to interfere with DC movement triggered by a secondary stimulus (BCG). Interestingly, conditioning with i-VACV doubled the number of migratory skin DCs reaching the dLN without enhancing the entry of BCG. We speculate that migration elicited by inactivated virus depleted skin DC pools available for BCG transport, contributing, in turn, to the delayed priming of BCG-specific CD4^+^ T cells noted in this setting.

VACV also blocked skin DC migration in response to a subsequent challenge with zymosan (Supplemental Fig. 2A), suggesting that this VACV-inhibitory effect is a more general phenomenon of skin infection with VACV. That said, this does not apply broadly to viral infections in the skin. There is a large body of data on the enveloped DNA viruses HSV-1 and HSV-2 showing that they trigger DC migration from skin (HSV-1) or the vaginal mucosa (HSV-2) to the dLN (36–39). Skin DC migration is also observed in response to the enveloped RNA viruses Semliki Forest virus and West Nile virus and the nonenveloped DNA virus adenovirus (40, 41). In these studies, DC migration was investigated mainly by FITC skin painting. We also confirm the ability of HSV-1 to trigger skin DC migration to the dLN in our footpad infection model that uses a CFSE injection-based migration assay (Supplemental Fig. 2B). VACV conditioning also blocked HSV-1–triggered DC migration (Supplemental Fig. 2B), in line with the VACV-inhibitory effect being a more general phenomenon.

Interestingly, respiratory infection with VACV has been shown to mobilize lung DCs to the dLN, and these lung-migratory DCs can also prime CD8^+^ T cells (42). Our study focused on DC migration in skin and, as such, did not investigate migration at other sites. Similar observations of bona fide DC migration from lung to the dLN have nevertheless been made in response to influenza virus (42–47), respiratory syncytial virus (48), and Sendai virus (49), supporting lung DC migration during virus respiratory infections. Thus, we propose that the DC migration-blocking effect of VACV in our model is a general phenomenon of VACV infection targeted to the skin. This is an important consideration for the future use of VACV vectors in vaccines and Ag delivery applications.

Enhanced mRNA accumulation of TNF-α, IL-1, and CCR7 in the skin was accompanied by skin DC migration in response to BCG but not VACV. Infection with VACV deletion mutants ΔA49, ΔB13, and ΔB15, which lack molecules that inhibit NF-ĸB, caspase-1, and IL-1, respectively, did to provoke DC migration (Supplemental Fig. 3), revealing, at least, that deletion of these specific molecules from VACV could not rescue the migration phenotype observed in our model. The contribution of IL-1R signaling to BCG-triggered migration is partial (3), so additional factors must regulate this process. During *M. tuberculosis* infection, IFN-α/β has been shown to block IL-1 production from myeloid cells (50). Whether the lack of IL-1 expression in VACV-infected skin or VACV inhibition of skin DC migration is coupled to IFN-α/β remains to be determined. Evaluating cytokine expression and DC migration in IFN-α/βR^−/−^ mice may help clarify this point.

Both i-VACV and MVA promoted migration of skin DC subsets that also relocate in response to BCG, but to our surprise, neither virus triggered the inflammatory transcription profile seen with BCG in the skin. It is possible that i-VACV and MVA trigger skin DC migration by different pathways. Indeed, enhanced CCR7 mRNA accumulation was observed with BCG and MVA but not with i-VACV. CCR7 expression is not an obligatory predictor of DC migration, and both CXCR4 and CXCR5 have been reported to guide DCs from the skin to the dLN (51, 52). Also, accumulation of migratory skin DCs in the dLN is not inhibited by footpad infection with the mouse-pathogenic poxvirus ectromelia (53), suggesting that blockade of DC migration by orthopoxviruses cannot be explained by virulence alone.

Although VACV did not induce transcription of proinflammatory cytokines in the skin, it did unleash a profound inflammatory infiltrate in the dLN. CD169^+^ subcapsular sinus (SCS) macrophages are directly exposed to afferent lymph-borne particulates and thus form a strategic line of defense in the dLN against free-flowing viruses, including VACV, preventing systemic viral spread (54). Previous studies confirm VACV infection of SCS macrophages (9, 55). In addition, MVA triggers inflammasome activation in SCS macrophages that leads to the recruitment of inflammatory cells into the LN (56). We observed an expansion of MHC-II^+^ CD11c^high^ CD11b^high^ cells expressing CD64 in VACV-infected dLN. Although MHC-II^+^ CD11c^high^ cells are generally recognized as LN-resident DCs, a network of macrophages with similar markers has recently been described in the LN paracortex during steady state (57). CD64^+^ DCs have also been observed in the dLN during *Listeria* infection, where they expand in response to IFN-α/β (58).

Migratory skin DCs are tasked with the transport of microbes and their Ags to the dLN and thus play a central role in priming T cells in the dLN postinfection or vaccination in the skin. In our study, VACV reached the dLN without mobilizing skin DCs, whose migration was blocked by the virus. Using a novel recombinant VACV, rVACV-Ag85B, we confirmed using P25 TCRTg cells that the virus is still clearly capable of priming Ag-specific CD4^+^ T cells in the dLN. This provides evidence for migratory skin DC-independent priming of CD4^+^ T cells during vaccination with a live attenuated vaccine. We speculate that DC-independent virus relocation occurs by VACV gaining direct access to lymphatic vessels. Previous studies show that VACV appear in the dLN within a few minutes after injection in the skin (9–12). We also report the virus in the pLN just minutes after inoculation in the footpad. This rapid relocation of virus to the dLN is in favor of direct viral access to lymphatics after skin infection. The rVACV-Ag85B developed for our studies may be a useful tool for mechanistic dissection of CD4^+^ T cell responses to heterologous Ags in the context of VACV vaccination. Indeed, the fate of DC transport-independent VACV in the LN paracortex, its interactions with APCs at this site, and the quality of ensuing CD4^+^ T cell priming are topics that merit further investigation and are likely to contribute to the development of novel vaccine-based counter-measures against infection and cancer.

## Supplementary Material

Data Supplement

## References

[1] Worbs, T. S. I. Hammerschmidt R. Förster 2017 Dendritic cell migration in health and disease. Nat. Rev. Immunol. 17: 30–48. 27890914 10.1038/nri.2016.116

[2] Randolph, G. J. V. Angeli M. A. Swartz 2005 Dendritic-cell trafficking to lymph nodes through lymphatic vessels. Nat. Rev. Immunol. 5: 617–628. 16056255 10.1038/nri1670

[3] Bollampalli, V. P. L. Harumi Yamashiro X. Feng D. Bierschenk Y. Gao H. Blom B. Henriques-Normark S. Nylén A. G. Rothfuchs 2015 BCG skin infection triggers IL-1R-MyD88-dependent migration of EpCAMlow CD11bhigh skin dendritic cells to draining lymph node during CD4+ T-cell priming. PLoS Pathog. 11: e1005206. 26440518 10.1371/journal.ppat.1005206 PMC4594926

[4] Mackett, M. G. L. Smith B. Moss 1982 Vaccinia virus: a selectable eukaryotic cloning and expression vector. Proc. Natl. Acad. Sci. USA 79: 7415–7419. 6296831 10.1073/pnas.79.23.7415 PMC347350

[5] Panicali, D. E. Paoletti 1982 Construction of poxviruses as cloning vectors: insertion of the thymidine kinase gene from herpes simplex virus into the DNA of infectious vaccinia virus. Proc. Natl. Acad. Sci. USA 79: 4927–4931. 6289324 10.1073/pnas.79.16.4927 PMC346798

[6] Perkus, M. E. A. Piccini B. R. Lipinskas E. Paoletti 1985 Recombinant vaccinia virus: immunization against multiple pathogens. Science 229: 981–984. 2992092 10.1126/science.2992092

[7] Moss, B. 1991 Vaccinia virus: a tool for research and vaccine development. Science 252: 1662–1667. 2047875 10.1126/science.2047875

[8] Ndiaye, B. P. F. Thienemann M. Ota B. S. Landry M. Camara S. Dièye T. N. Dieye H. Esmail R. Goliath K. Huygen MVA85A 030 trial investigators 2015 Safety, immunogenicity, and efficacy of the candidate tuberculosis vaccine MVA85A in healthy adults infected with HIV-1: a randomised, placebo-controlled, phase 2 trial. Lancet Respir. Med. 3: 190–200. 25726088 10.1016/S2213-2600(15)00037-5 PMC4648060

[9] Norbury, C. C. D. Malide J. S. Gibbs J. R. Bennink J. W. Yewdell 2002 Visualizing priming of virus-specific CD8+ T cells by infected dendritic cells in vivo. Nat. Immunol. 3: 265–271. 11828323 10.1038/ni762

[10] Hickman, H. D. K. Takeda C. N. Skon F. R. Murray S. E. Hensley J. Loomis G. N. Barber J. R. Bennink J. W. Yewdell 2008 Direct priming of antiviral CD8+ T cells in the peripheral interfollicular region of lymph nodes. Nat. Immunol. 9: 155–165. 18193049 10.1038/ni1557

[11] Kastenmüller, W. P. Torabi-Parizi N. Subramanian T. Lämmermann R. N. Germain 2012 A spatially-organized multicellular innate immune response in lymph nodes limits systemic pathogen spread. Cell 150: 1235–1248. 22980983 10.1016/j.cell.2012.07.021 PMC3514884

[12] Reynoso, G. V. A. S. Weisberg J. P. Shannon D. T. McManus L. Shores J. L. Americo R. V. Stan J. W. Yewdell H. D. Hickman 2019 Lymph node conduits transport virions for rapid T cell activation. Nat. Immunol. 20: 602–612. 30886418 10.1038/s41590-019-0342-0 PMC6474694

[13] Hickman, H. D. G. V. Reynoso B. F. Ngudiankama E. J. Rubin J. G. Magadán S. S. Cush J. Gibbs B. Molon V. Bronte J. R. Bennink J. W. Yewdell 2013 Anatomically restricted synergistic antiviral activities of innate and adaptive immune cells in the skin. Cell Host Microbe 13: 155–168. 23414756 10.1016/j.chom.2013.01.004 PMC3591514

[14] Khan, T. N. J. L. Mooster A. M. Kilgore J. F. Osborn J. C. Nolz 2016 Local antigen in nonlymphoid tissue promotes resident memory CD8+ T cell formation during viral infection. J. Exp. Med. 213: 951–966. 27217536 10.1084/jem.20151855 PMC4886364

[15] Loo, C. P. N. A. Nelson R. S. Lane J. L. Booth S. C. Loprinzi Hardin A. Thomas M. K. Slifka J. C. Nolz A. W. Lund 2017 Lymphatic vessels balance viral dissemination and immune activation following cutaneous viral infection. Cell Rep. 20: 3176–3187. 28954233 10.1016/j.celrep.2017.09.006 PMC5621787

[16] Smith, G. L. C. T. O. Benfield C. Maluquer de Motes M. Mazzon S. W. J. Ember B. J. Ferguson R. P. Sumner 2013 Vaccinia virus immune evasion: mechanisms, virulence and immunogenicity. J. Gen. Virol. 94: 2367–2392. 23999164 10.1099/vir.0.055921-0

[17] Egen, J. G. A. G. Rothfuchs C. G. Feng M. A. Horwitz A. Sher R. N. Germain 2011 Intravital imaging reveals limited antigen presentation and T cell effector function in mycobacterial granulomas. Immunity 34: 807–819. 21596592 10.1016/j.immuni.2011.03.022 PMC3164316

[18] Rothfuchs, A. G. J. G. Egen C. G. Feng L. R. Antonelli A. Bafica N. Winter R. M. Locksley A. Sher 2009 *In situ* IL-12/23p40 production during mycobacterial infection is sustained by CD11b^high^ dendritic cells localized in tissue sites distinct from those harboring bacilli. J. Immunol. 182: 6915–6925. 19454688 10.4049/jimmunol.0900074 PMC2786988

[19] Mansur, D. S. C. Maluquer de Motes L. Unterholzner R. P. Sumner B. J. Ferguson H. Ren P. Strnadova A. G. Bowie G. L. Smith 2013 Poxvirus targeting of E3 ligase β-TrCP by molecular mimicry: a mechanism to inhibit NF-κB activation and promote immune evasion and virulence. PLoS Pathog. 9: e1003183. 23468625 10.1371/journal.ppat.1003183 PMC3585151

[20] Kettle, S. N. W. Blake K. M. Law G. L. Smith 1995 Vaccinia virus serpins B13R (SPI-2) and B22R (SPI-1) encode M(r) 38.5 and 40K, intracellular polypeptides that do not affect virus virulence in a murine intranasal model. Virology 206: 136–147. 7831769 10.1016/s0042-6822(95)80028-x

[21] Alcamí, A. G. L. Smith 1992 A soluble receptor for interleukin-1 beta encoded by vaccinia virus: a novel mechanism of virus modulation of the host response to infection. Cell 71: 153–167. 1394428 10.1016/0092-8674(92)90274-g

[22] Cotter, C. A. P. L. Earl L. S. Wyatt B. Moss 2017 Preparation of cell cultures and vaccinia virus stocks. Curr. Protoc. Protein Sci. 89: 5.12.1–5.12.18. 10.1002/cpps.34 28762495

[23] Falkner, F. G. B. Moss 1988 *Escherichia coli* gpt gene provides dominant selection for vaccinia virus open reading frame expression vectors. J. Virol. 62: 1849–1854. 3130492 10.1128/jvi.62.6.1849-1854.1988 PMC253265

[24] Falkner, F. G. B. Moss 1990 Transient dominant selection of recombinant vaccinia viruses. J. Virol. 64: 3108–3111. 2159565 10.1128/jvi.64.6.3108-3111.1990 PMC249504

[25] Bollampalli, V. P. S. Nylén A. G. Rothfuchs 2016 A CFSE-based assay to study the migration of murine skin dendritic cells into draining lymph nodes during infection with *Mycobacterium bovis* Bacille Calmette-Guérin. J. Vis. Exp. 116: 54620. 10.3791/54620 PMC5092184 27768071

[26] Tsung, K. J. H. Yim W. Marti R. M. Buller J. A. Norton 1996 Gene expression and cytopathic effect of vaccinia virus inactivated by psoralen and long-wave UV light. J. Virol. 70: 165–171. 8523521 10.1128/jvi.70.1.165-171.1996 PMC189801

[27] Sancho, M. C. S. Schleich G. Griffiths J. Krijnse-Locker 2002 The block in assembly of modified vaccinia virus Ankara in HeLa cells reveals new insights into vaccinia virus morphogenesis. J. Virol. 76: 8318–8334. 12134037 10.1128/JVI.76.16.8318-8334.2002 PMC155139

[28] Obieglo, K. X. Feng V. P. Bollampalli I. Dellacasa-Lindberg C. Classon M. Österblad H. Helmby J. P. Hewitson R. M. Maizels A. Gigliotti Rothfuchs S. Nylén 2016 Chronic gastrointestinal nematode infection mutes immune responses to mycobacterial infection distal to the gut. J. Immunol. 196: 2262–2271. 26819205 10.4049/jimmunol.1500970 PMC4760231

[29] Sandgren, K. J. J. Wilkinson M. Miranda-Saksena G. M. McInerney K. Byth-Wilson P. J. Robinson A. L. Cunningham 2010 A differential role for macropinocytosis in mediating entry of the two forms of vaccinia virus into dendritic cells. PLoS Pathog. 6: e1000866. 20421949 10.1371/journal.ppat.1000866 PMC2858709

[30] Yates, N. L. M. A. Alexander-Miller 2007 Vaccinia virus infection of mature dendritic cells results in activation of virus-specific naïve CD8+ T cells: a potential mechanism for direct presentation. Virology 359: 349–361. 17056088 10.1016/j.virol.2006.09.020 PMC1861836

[31] Chahroudi, A. D. A. Garber P. Reeves L. Liu D. Kalman M. B. Feinberg 2006 Differences and similarities in viral life cycle progression and host cell physiology after infection of human dendritic cells with modified vaccinia virus Ankara and vaccinia virus. J. Virol. 80: 8469–8481. 16912297 10.1128/JVI.02749-05 PMC1563888

[32] Jenne, L. C. Hauser J. F. Arrighi J. H. Saurat A. W. Hügin 2000 Poxvirus as a vector to transduce human dendritic cells for immunotherapy: abortive infection but reduced APC function. Gene Ther. 7: 1575–1583. 11021596 10.1038/sj.gt.3301287

[33] Engelmayer, J. M. Larsson M. Subklewe A. Chahroudi W. I. Cox R. M. Steinman N. Bhardwaj 1999 Vaccinia virus inhibits the maturation of human dendritic cells: a novel mechanism of immune evasion. J. Immunol. 163: 6762–6768. 10586075

[34] Yao, Y. P. Li P. Singh A. T. Thiele D. S. Wilkes G. J. Renukaradhya R. R. Brutkiewicz J. B. Travers G. D. Luker S. C. Hong 2007 Vaccinia virus infection induces dendritic cell maturation but inhibits antigen presentation by MHC class II. Cell. Immunol. 246: 92–102. 17678637 10.1016/j.cellimm.2007.06.005 PMC2100387

[35] Humrich, J. Y. P. Thumann S. Greiner J. H. Humrich M. Averbeck C. Schwank E. Kämpgen G. Schuler L. Jenne 2007 Vaccinia virus impairs directional migration and chemokine receptor switch of human dendritic cells. Eur. J. Immunol. 37: 954–965. 17357104 10.1002/eji.200636230

[36] Bedoui, S. P. G. Whitney J. Waithman L. Eidsmo L. Wakim I. Caminschi R. S. Allan M. Wojtasiak K. Shortman F. R. Carbone 2009 Cross-presentation of viral and self antigens by skin-derived CD103+ dendritic cells. Nat. Immunol. 10: 488–495. 19349986 10.1038/ni.1724

[37] Lee, H. K. M. Zamora M. M. Linehan N. Iijima D. Gonzalez A. Haberman A. Iwasaki 2009 Differential roles of migratory and resident DCs in T cell priming after mucosal or skin HSV-1 infection. J. Exp. Med. 206: 359–370. 19153243 10.1084/jem.20080601 PMC2646574

[38] Allan, R. S. J. Waithman S. Bedoui C. M. Jones J. A. Villadangos Y. Zhan A. M. Lew K. Shortman W. R. Heath F. R. Carbone 2006 Migratory dendritic cells transfer antigen to a lymph node-resident dendritic cell population for efficient CTL priming. Immunity 25: 153–162. 16860764 10.1016/j.immuni.2006.04.017

[39] Zhao, X. E. Deak K. Soderberg M. Linehan D. Spezzano J. Zhu D. M. Knipe A. Iwasaki 2003 Vaginal submucosal dendritic cells, but not Langerhans cells, induce protective Th1 responses to herpes simplex virus-2. J. Exp. Med. 197: 153–162. 12538655 10.1084/jem.20021109 PMC2193810

[40] Bachy, V. C. Hervouet P. D. Becker L. Chorro L. M. Carlin S. Herath T. Papagatsias J. B. Barbaroux S. J. Oh A. Benlahrech 2013 Langerin negative dendritic cells promote potent CD8+ T-cell priming by skin delivery of live adenovirus vaccine microneedle arrays. Proc. Natl. Acad. Sci. USA 110: 3041–3046. 23386724 10.1073/pnas.1214449110 PMC3581957

[41] Johnston, L. J. G. M. Halliday N. J. King 2000 Langerhans cells migrate to local lymph nodes following cutaneous infection with an arbovirus. J. Invest. Dermatol. 114: 560–568. 10692118 10.1046/j.1523-1747.2000.00904.x

[42] Beauchamp, N. M. R. Y. Busick M. A. Alexander-Miller 2010 Functional divergence among CD103+ dendritic cell subpopulations following pulmonary poxvirus infection. J. Virol. 84: 10191–10199. 20660207 10.1128/JVI.00892-10 PMC2937786

[43] Kandasamy, M. P. C. Ying A. W. Ho H. R. Sumatoh A. Schlitzer T. R. Hughes D. M. Kemeny B. P. Morgan F. Ginhoux B. Sivasankar 2013 Complement mediated signaling on pulmonary CD103(+) dendritic cells is critical for their migratory function in response to influenza infection. PLoS Pathog. 9: e1003115. 23326231 10.1371/journal.ppat.1003115 PMC3542115

[44] Ho, A. W. N. Prabhu R. J. Betts M. Q. Ge X. Dai P. E. Hutchinson F. C. Lew K. L. Wong B. J. Hanson P. A. Macary D. M. Kemeny 2011 Lung CD103+ dendritic cells efficiently transport influenza virus to the lymph node and load viral antigen onto MHC class I for presentation to CD8 T cells. J. Immunol. 187: 6011–6021. 22043017 10.4049/jimmunol.1100987

[45] Ballesteros-Tato, A. B. León F. E. Lund T. D. Randall 2010 Temporal changes in dendritic cell subsets, cross-priming and costimulation via CD70 control CD8(+) T cell responses to influenza. Nat. Immunol. 11: 216–224. 20098442 10.1038/ni.1838 PMC2822886

[46] Belz, G. T. C. M. Smith L. Kleinert P. Reading A. Brooks K. Shortman F. R. Carbone W. R. Heath 2004 Distinct migrating and nonmigrating dendritic cell populations are involved in MHC class I-restricted antigen presentation after lung infection with virus. Proc. Natl. Acad. Sci. USA 101: 8670–8675. 15163797 10.1073/pnas.0402644101 PMC423253

[47] Legge, K. L. T. J. Braciale 2003 Accelerated migration of respiratory dendritic cells to the regional lymph nodes is limited to the early phase of pulmonary infection. Immunity 18: 265–277. 12594953 10.1016/s1074-7613(03)00023-2

[48] Lukens, M. V. D. Kruijsen F. E. Coenjaerts J. L. Kimpen G. M. van Bleek 2009 Respiratory syncytial virus-induced activation and migration of respiratory dendritic cells and subsequent antigen presentation in the lung-draining lymph node. J. Virol. 83: 7235–7243. 19420085 10.1128/JVI.00452-09 PMC2704789

[49] Grayson, M. H. M. S. Ramos M. M. Rohlfing R. Kitchens H. D. Wang A. Gould E. Agapov M. J. Holtzman 2007 Controls for lung dendritic cell maturation and migration during respiratory viral infection. J. Immunol. 179: 1438–1448. 17641009 10.4049/jimmunol.179.3.1438

[50] Mayer-Barber, K. D. B. B. Andrade D. L. Barber S. Hieny C. G. Feng P. Caspar S. Oland S. Gordon A. Sher 2011 Innate and adaptive interferons suppress IL-1α and IL-1β production by distinct pulmonary myeloid subsets during Mycobacterium tuberculosis infection. Immunity 35: 1023–1034. 22195750 10.1016/j.immuni.2011.12.002 PMC3246221

[51] Kabashima, K. N. Shiraishi K. Sugita T. Mori A. Onoue M. Kobayashi J. Sakabe R. Yoshiki H. Tamamura N. Fujii 2007 CXCL12-CXCR4 engagement is required for migration of cutaneous dendritic cells. Am. J. Pathol. 171: 1249–1257. 17823289 10.2353/ajpath.2007.070225 PMC1988874

[52] Saeki, H. M. T. Wu E. Olasz S. T. Hwang 2000 A migratory population of skin-derived dendritic cells expresses CXCR5, responds to B lymphocyte chemoattractant *in vitro*, and co-localizes to B cell zones in lymph nodes *in vivo.* Eur. J. Immunol. 30: 2808–2814. 11069061 10.1002/1521-4141(200010)30:10<2808::AID-IMMU2808>3.0.CO;2-K

[53] Stotesbury, C. E. B. Wong L. Tang B. Montoya C. J. Knudson C. R. Melo-Silva L. J. Sigal 2020 Defective early innate immune response to ectromelia virus in the draining lymph nodes of aged mice due to impaired dendritic cell accumulation. Aging Cell 19: e13170. 32657004 10.1111/acel.13170 PMC7433008

[54] Louie, D. A. P. S. Liao 2019 Lymph node subcapsular sinus macrophages as the frontline of lymphatic immune defense. Front. Immunol. 10: 347. 30891035 10.3389/fimmu.2019.00347 PMC6413714

[55] Tian, T. M. Q. Jin K. Dubin S. L. King W. Hoetzenecker G. F. Murphy C. A. Chen T. S. Kupper R. C. Fuhlbrigge 2017 IL-1R type 1-deficient mice demonstrate an impaired host immune response against cutaneous vaccinia virus infection. J. Immunol. 198: 4341–4351. 28468973 10.4049/jimmunol.1500106 PMC5506850

[56] Sagoo, P. Z. Garcia B. Breart F. Lemaître D. Michonneau M. L. Albert Y. Levy P. Bousso 2016 In vivo imaging of inflammasome activation reveals a subcapsular macrophage burst response that mobilizes innate and adaptive immunity. Nat. Med. 22: 64–71. 26692332 10.1038/nm.4016

[57] Baratin, M. L. Simon A. Jorquera C. Ghigo D. Dembele J. Nowak R. Gentek S. Wienert F. Klauschen B. Malissen 2017 T cell zone resident macrophages silently dispose of apoptotic cells in the lymph node. Immunity 47: 349–362.e5. 28801233 10.1016/j.immuni.2017.07.019

[58] Min, J. D. Yang M. Kim K. Haam A. Yoo J.-H. Choi B. U. Schraml Y. S. Kim D. Kim S.-J. Kang 2018 Inflammation induces two types of inflammatory dendritic cells in inflamed lymph nodes. [Published erratum appears in 2018 *Exp. Mol. Med.* 50: 33.] Exp. Mol. Med. 50: e458. 29546878 10.1038/emm.2017.292 PMC5898896

