# Supplementary material for: Vaccinia Virus Infection Inhibits Skin Dendritic Cell Migration to the Draining Lymph Node
Source: J Immunol. 2021 Jan 8;206(4):776–84. doi: 10.4049/jimmunol.2000928 (PMC7851745; doi:10.4049/jimmunol.2000928)
Supplement: Data Supplement [file JI_2000928.zip › JI_2000928_Supplemental_Figures_1.pdf]

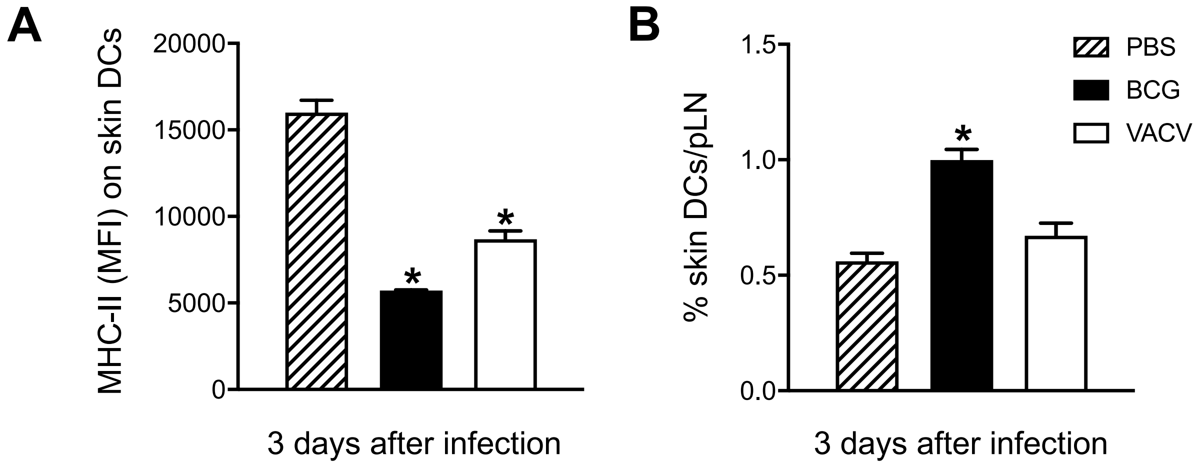

SUPPLEMENTARY FIGURE 1. Expression of MHC-II and frequency of skin DCs in dLN of VACV- and BCG-infected mice. (A and B) C57BL/6 mice were inoculated in the footpad skin with PBS, BCG (10^6 CFUs) or VACV (10^6 PFUs). Three days later, pLNs were processed and analyzed by flow cytometry. (A) Mean fluorescence intensity (MFI) for MHC-II on skin DCs in pLN. (B) Frequency of skin DCs in pLN. Five animals per group used in each experiment. One of 3 independent experiment shown. Bars indicate standard error of the mean. * denotes statistically significant difference between PBS and infected groups (A) and PBS and BCG (B).

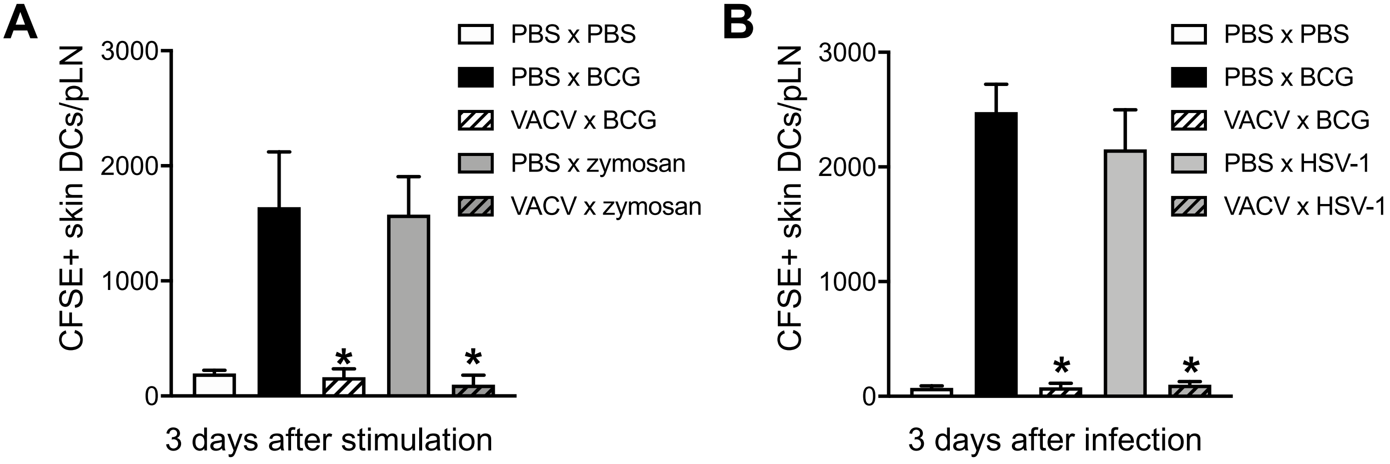

SUPPLEMENTARY FIGURE 2. Conditioning with VACV mutes subsequent DC migration in response to zymosan and HSV-1. C57BL/6 mice were inoculated in the footpad skin with PBS or VACV (10^6 PFUs). Twenty-four hours later the same footpads were inoculated with BCG (10^6 CFUs) (A and B), zymosan (100 µg) (A) or HSV-1 (10^5 PFUs) (B) and the CFSE-based migration assay performed as in Fig. 1. Total number of CFSE-labeled skin DCs in the pLN 3 days after stimulation/infection are shown. Four to 5 animals per group were used. Bars indicate standard error of the mean. One of 3 independent experiments for zymosan. One of 2 independent experiments for HSV-1. * denotes statistical significance between PBS- and VACV-conditioned groups. PBS x BCG, PBS x zymosan and PBS x HSV-1 groups are statistically significant from PBS x PBS controls.

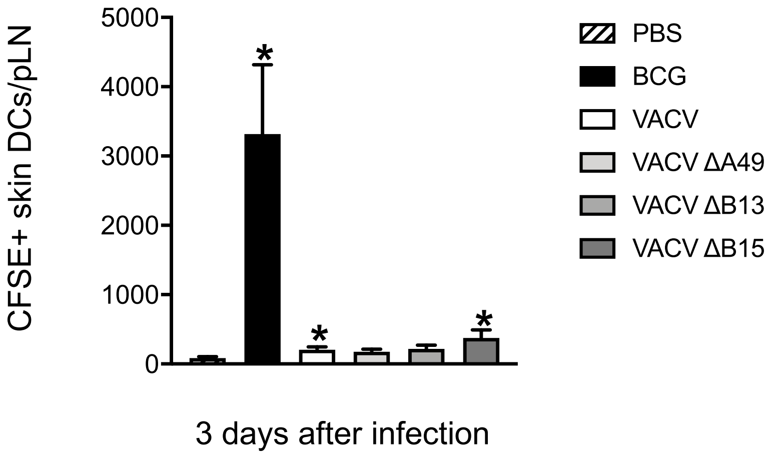

SUPPLEMENTARY FIGURE 3. Infection with VACV ΔA49, ΔB13 and ΔB15 does not trigger skin DC migration to dLN. C57BL/6 mice were inoculated in the footpad skin with PBS, BCG (10^6 CFUs), VACV (10^6 PFUs) or the VACV deletion mutants ΔA49, ΔB13 and ΔB15 (10^6 PFUs) and subjected to the CFSE migration assay as in Fig. 1. Total number of CFSE-labeled skin DCs in the pLN 3 days after infection are shown. Five animals per group were used. Bars indicate standard error of the mean. * denotes statistical significance between PBS- and vaccine-injected groups.
